# Advances in research on the correlation between LTCCBs and cardiovascular diseases: A review

**DOI:** 10.1097/MD.0000000000042799

**Published:** 2025-06-20

**Authors:** Xiao Yang, Xinghui Li

**Affiliations:** a The First Clinical Medical College of Gansu University of Traditional Chinese Medicine, Lanzhou City, Gansu Province, People’s Republic of China; b Department of Cardiology, Gansu Provincial People’s Hospital, Lanzhou City, Gansu Province, People’s Republic of China.

**Keywords:** CCBs, CVDs, LTCCBs, LTCCs

## Abstract

The prevalence of cardiovascular diseases has been rising globally, and various therapies have made significant strides in controlling the progression of these conditions and improving prognosis. Among these, pharmacological therapies have evolved rapidly and are increasingly integrated into clinical practice. Pharmacological treatments play a pivotal role in both basic research and clinical applications, particularly in deriving accurate conclusions from drug-related trials to address clinical challenges in drug selection and improve the cure rates of various diseases. L-type calcium channel blockers (LTCCBs) have attracted considerable research attention in recent years. With the accelerated development of novel drugs, the range of LTCCBs-based drug types has also diversified. This review aims to explore the relationship between LTCCBs and conditions such as hypertension, arrhythmias, ischemia–reperfusion injury, and cardiomyopathy, with the goal of contributing to the diversification of treatment options for cardiovascular diseases patients. Additionally, it seeks to establish a theoretical foundation for future research and the clinical application of these therapies. It is imperative to conduct a thorough analysis and in-depth discussion regarding the deficiencies in the medication process as well as various application scenarios. With the advancement of science and the introduction of novel drugs into clinical practice, it is anticipated that these developments will play a critical guiding role in the diagnosis and treatment of complex clinical diseases.

## 1. Introduction

Calcium channel blockers (CCBs) are a diverse group of drugs in terms of both structure and function, and they can be classified into dihydropyridines (e.g., nifedipine) and non-dihydropyridines (e.g., verapamil).^[1]^ One of the recent research hotspots is the selective action of L-type calcium channel blockers (LTCCBs) on L-type Ca^2+^ channels (LTCCs). Cardiovascular diseases (CVDs), including coronary artery disease, remain the leading cause of mortality and disease burden globally. Social determinants such as population growth, aging, and urbanization are key factors driving the continued increase in the burden of CVDs. Therefore, it is crucial to strengthen primary prevention and health management of CVDs.^[2]^ Increasing evidence suggests that LTCCBs-based drugs play an increasingly important role in the treatment of CVDs. However, the development of new drugs in this area is still in its early stages, highlighting the need to further elucidate their precise mechanisms of action and explore their potential for expanding therapeutic applications. Given the significant therapeutic potential of LTCCBs in disease treatment, this review aims to comprehensively analyze relevant research findings both domestically and internationally, providing valuable insights for clinical diagnosis and treatment.

## 2. LTCCBs

LTCCs provide the calcium influx necessary to trigger cardiac contraction in a graded manner, regulated by the amplitude and kinetics of the L-type calcium current (ICa-L). The inactivation of LTCCs is controlled by voltage-dependent inactivation and calcium-dependent inactivation, which finely tunes calcium flux.^[3]^ Normal ion channel activity is crucial for maintaining electrochemical balance and stable ion concentrations. Regarding the regulation of LTCCs activity, Sanchez-Alonso et al^[4]^ demonstrated that the regulation of LTCCs by adjacent coupling occurred solely through β2-adrenergic receptors, rather than β1-adrenergic receptors. Future research could further explore whether other receptor types may modulate different effects, thereby enhancing our understanding of the signaling pathways involved.

LTCCs, also known as Cav1, are voltage-gated calcium channels that play key roles in a wide range of physiological processes, including neurotransmission, muscle contraction, and gene expression. Dysregulation of calcium channels has been implicated in neurological, cardiovascular, muscular, and psychiatric disorders.^[5]^ Previous research has elucidated the classification of LTCCs, which includes the Cav1.2 and Cav1.3 subtypes, encoded by the *CACNA1C* and *CACNA1D* genes, respectively. These subtypes are critical regulators of calcium entry into neurons, mediating neurotransmitter release and synaptic plasticity.^[6]^ Additionally, preclinical evidence suggests that LTCCs may play a key role in the development of various forms of drug dependence, with mechanisms involving alterations in midbrain dopaminergic neuron activity, genomic effects, and changes in synaptic plasticity.^[7]^ Further neurophysiological studies are needed to clarify the relationship between neurotransmitter release mediated by LTCCs and their corresponding neuronal effects.

The regulation of specific biological processes also depends on the interplay between membrane potential changes and ion concentration shifts. For example, high-voltage-activated calcium channels (Cav1/Cav2) in excitable cells convert action potentials into Ca^2+^ influx, which in turn controls essential biological processes such as hormone secretion and activity-dependent gene expression. The regulation of Cav1/Cav2 channel activity is a powerful physiological mechanism, with various intracellular signaling molecules modulating different aspects of Cav channel function.^[8]^ Furthermore, research has revealed that Ca^2+^ influx through high-voltage-activated calcium channels controls multiple cellular functions, with subunit diversity being a key characteristic of how calcium signaling mediates distinct functional outcomes.^[9]^ Therefore, understanding the structure, classification, and functions of these subunits and their relationship with calcium influx is crucial for deepening our knowledge of channel transport function.

CCBs remain a cornerstone of pharmacotherapy for CVDs, with their effects demonstrated across a range of drugs. For example, nimodipine, a dihydropyridine CCBs, blocks the influx of extracellular calcium through L-type voltage-gated calcium channels.^[10]^ This suggests that future quantitative observations through related experiments could clarify the graded responses of calcium influx. Channel excitability is essential for normal function, both in the cardiovascular and nervous systems. Hyperpolarization-activated cyclic nucleotide-gated channels (subtypes 1–4) are widely expressed in the central and peripheral nervous systems, as well as in smooth muscle cells of various organs, where they primarily regulate the excitability of these tissues.^[11]^

## 3. LTCCBs and CVDs

### 3.1. Hypertension

In 2018, the European Society of Cardiology and the European Society of Hypertension proposed a conventional definition of hypertension, that is, BP ≥ 140/90 mm Hg.^[12]^ Elevated systemic arterial pressure is closely associated with increased morbidity and mortality from CVDs.^[13]^ Although awareness, treatment, and control rates of hypertension have improved, they remain at relatively low levels. As of 2021, China had 245 million hypertensive patients, imposing a growing economic burden on residents and society, making it a major public health issue.^[14]^ Since hypertension is a risk factor for many common diseases, effectively controlling blood pressure levels is crucial for ensuring the health of the general population.

A large body of evidence indicates that selecting appropriate drug combinations is crucial for controlling blood pressure. LTCCBs have been widely used as antihypertensive agents in clinical practice.^[15]^ The 1,4-dihydropyridine class of LTCCBs is the most important and is currently primarily used to treat CVDs, particularly hypertension.^[16–18]^ Changes in the physicochemical properties of the blood may be related to fluctuations in blood pressure. A study by Ito et al^[19]^ concluded that cilnidipine can reduce pre- and post-dialysis systolic blood pressure (SBP) and may also reduce intradialytic SBP, indicating that cilnidipine may effectively lower SBP in hypertensive patients undergoing dialysis. Additionally, Nasu et al,^[20]^ using HEK293 cells expressing the Cav1.2 channel, found that azelnidipine significantly reduced the surface expression of Cav1.2α1c, while other LTCCBs, such as amlodipine and nicardipine, did not exhibit this effect, which may partially explain its sustained antihypertensive action. This suggests that improving the alignment between drug selection and clinical indications could enhance therapeutic efficacy.

In recent years, many studies have focused on experimental research related to spontaneous hypertension, particularly in terms of blood pressure reduction. Wang et al,^[21]^ using Wistar–Kyoto rats and spontaneously hypertensive rats, found that hydroxysafflor yellow A can reduce intracellular free calcium ion levels and activate BKCa channels, thereby lowering intracellular free Ca^2+^ levels by inhibiting Ca-L channels. This may be a key mechanism underlying its vasodilation and blood pressure-lowering effects. In another study, Xu et al^[22]^ observed in animal experiments that hydrogen sulfide enhanced the baroreceptor reflex in isolated carotid sinuses, possibly by inhibiting LTCCs, providing a potential new target for hypertension prevention and treatment. Identifying experimental pathway targets based on ion channel opening and closing is necessary and may offer new directions for blood pressure research.

As research has progressed, studies on channel-regulating substances have gradually increased. In an experiment by Reyes et al,^[23]^ using a transgenic rat model that overexpresses the human angiotensinogen gene [TGR(hAGT)L1623], it was found that a calcium channel regulatory activity existed in cardiomyocytes. This activity responds to signaling via the intracellular receptor-mediated Ang-(1-12)/chymase axis, generating angiotensin II (Ang II), which increases intracellular calcium through ICa-L in TGR(hAGT)L1623 hearts. Thus, activation of the renin-angiotensin system by Ang-(1-12)/chymase may contribute to treating pathological phenotypes such as chronic hypertension. The antihypertensive effects of natural products are also noteworthy. Alsayed et al^[24]^ demonstrated that the antihypertensive effect of hibiscus was related to its vasodilatory action, which is independent of endothelial cells, suggesting that the reduction in ICa-L contributes to this effect. Therefore, the therapeutic efficacy of antihypertensive drugs is closely related to the vasodilatory and contractile actions of blood vessels. Yun et al^[25]^ explored the vasodilatory effects of 24 alkaloids (1–24) from Uncaria on phenylephrine-induced contractions of the mesenteric arteries in rats and found that hirsutine A (21) exerted its effects independently of endothelium-derived vasorelaxation factors. This suggests that hirsutine A (21) may be a potential LTCCBs for the effective treatment of hypertension, although its mechanism of action remains unclear and warrants further investigation. However, Johnson et al^[26]^ found that elderly patients or those with advanced hypertension and/or cardiovascular remodeling should use voltage-gated LTCCBs with caution due to elevated levels of matrix interaction molecules, Ca^2+^ sensor proteins, and plasma membranes in these patients. Therefore, larger animal studies and clinical trials are needed to further validate these findings and better guide clinical practice.

### 3.2. Arrhythmias

Arrhythmias typically refer to disturbances in the rate or rhythm of the heartbeat. Sinoatrial node cardiomyocytes exhibit automatic and rhythmic electrical activity, and their rate is influenced by inputs from the autonomic nervous system. For instance, sympathetic nerves increase heart rate by activating the β-adrenergic receptor (β-ARs) signaling cascade, and LTCCs activity facilitates membrane depolarization, serving as a central target of β-ARs signaling.^[27]^ Atrial fibrillation is one of the most common types of arrhythmia, and over the past few decades, more than 160 genes have been associated with atrial fibrillation, some discovered through classical linkage studies, while most were identified through functional studies or genome-wide association studies.^[28]^ The progression of arrhythmias is often closely related to changes in ion concentrations at the microscopic level. For example, acute changes in Ca^2+^ influx through Cav1.2 channels can result in arrhythmias and sudden cardiac death.^[29]^

The development of drugs based on plant extracts has become a research hotspot in recent years. Liu et al,^[30]^ using whole-cell patch-clamp techniques, investigated the effects of isoliensinine (IL), an alkaloid extracted from lotus seeds, on transmembrane ion currents and action potentials in isolated rabbit left ventricular myocytes. The study showed that IL effectively reduced depolarization in ventricular myocytes by inhibiting late sodium currents and ICa-Ls, suggesting that IL has potential anti-arrhythmic properties. Additionally, Angelini et al^[31]^ extended their research from human CaV1.2 channel clones to rabbit ventricular myocytes and perfused hearts from both rats and rabbits, demonstrating that roscovitine selectively reduced the non-inactivating component of ICa,L (late-phase Ca^2+^ influx during the action potential) in human CaV1.2 clones and ventricular myocytes. This finding supports the strategy of selectively reducing late ICa,L as an anti-arrhythmic approach, suggesting that anti-arrhythmic therapies should focus on modifying the gating properties of CaV1.2 channels. Thus, both IL and roscovitine offer a promising foundation for the development of anti-arrhythmic drugs in the future.

Supraventricular tachycardia (SVT) is a common cause of hospital admissions and can cause significant discomfort and distress in patients. The most common types include atrioventricular (AV) nodal reentrant tachycardia, AV reentrant tachycardia, and atrial tachycardia.^[32]^ James et al^[33]^ evaluated the efficacy and safety of an experimental fast-acting non-dihydropyridine LTCCBs, etripamil, in a nasal spray formulation, for terminating symptomatic, persistent paroxysmal SVT. Their findings concluded that etripamil effectively terminated the condition, and self-administration of the drug during episodes was safe and well-tolerated. Additionally, the dosage of the drug is an important factor that cannot be overlooked. Eroglu et al^[34]^ conducted a case–control study that demonstrated an association between high-dose nifedipine and an increased risk of out-of-hospital cardiac arrest in the general population, while low-dose nifedipine or any dose of amlodipine was not associated with this risk. Therefore, careful attention to drug dosage is crucial for optimizing therapeutic efficacy, especially in populations at risk for specific disease conditions.

### 3.3. Ischemia–reperfusion (IR) injury

IR injury is a pathological process that occurs in multiple organs throughout the body, often associated with severe cellular damage and death.^[35]^ Oxidative stress and the accompanying inflammatory response induced by IR can lead to increased morbidity and mortality in conditions such as acute coronary syndromes, stroke, and limb injuries. Ischemia results in severe hypoxia and tissue dysfunction, while subsequent reperfusion exacerbates tissue injury by inducing cell death and activating inflammatory responses.^[36]^ Various types of cell death, including necrosis, apoptosis, and autophagy, can occur during the IR injury process.^[37]^

A considerable number of studies have demonstrated that different drugs of the same class can exert varying degrees of efficacy in specific conditions. Nessa et al^[38]^ compared the effects of cilnidipine and amlodipine on post-infarction left ventricular remodeling in spontaneously hypertensive rats, concluding that cilnidipine reduced cardiac norepinephrine concentration and inhibited the renin-angiotensin system, thus mitigating post-myocardial infarction remodeling more effectively than amlodipine. Wang et al^[39]^ demonstrated in rat experiments that autophagy inhibitors negatively regulated cardiomyocyte contraction rhythm and field potential signals. They found that Ad-BAG3 inhibited autophagy by modulating the expression of autophagy-related proteins, while treatment with autophagy agonists reduced calcium overload. Calenduloside E promoted autophagy through BAG3, regulated LTCCs expression, inhibited calcium overload, and ultimately alleviated myocardial IR injury. This suggests that linking intermediate metabolic pathways could establish a mechanistic relationship between LTCCBs and myocardial IR injury.

Quercetin has been identified in recent years as a factor with significant therapeutic potential for myocardial IR injury. Liang et al,^[40]^ using a myocardial ischemia animal model established by subcutaneous injection of isoproterenol and isolated rat cardiomyocytes, found that quercetin decreased ICa-L in a concentration-dependent manner. Quercetin also shifted the current–voltage curve upward, and both the activation and inactivation curves shifted to the left. Furthermore, quercetin inhibited the amplitude of cell shortening and Ca^2+^ transients, suggesting that quercetin, as an LTCCs inhibitor, exerts cardioprotective effects by inhibiting Ca^2+^ influx and myocardial contractility. However, the precise mechanism underlying these effects remains unclear, making it a focal point for future research. Additionally, andrographolide has also been identified as a promising factor for further investigation. Elasoru et al^[41]^ demonstrated in animal studies that andrographolide could serve as a potential therapeutic agent against early subacute myocardial infarction and could be developed as a template for semi-synthetic drugs for myocardial protection. Thus, exploring the inverse properties of existing drugs to develop novel therapeutic agents holds significant promise. Combining modern instrumentation and precise measurement of key data will enhance the accuracy of comparisons and promote the development of new drugs.

### 3.4. Cardiomyopathy

Significant progress has been made in the taxonomy of myocardial diseases over the past 25 years, with new cardiomyopathies, such as arrhythmogenic, restrictive, and non-compaction cardiomyopathies, being discovered and added to the updated World Health Organization classification.^[42]^ Rare genetic variants play a major role in the pathogenesis of cardiomyopathies, which are empirically considered monogenic diseases. It has also been elucidated that the clinical course of cardiomyopathies varies depending on the pathogenic gene.^[43]^ Current studies reveal the existence of some disease phenomena, but extensive basic and clinical research is still required to accelerate the clarification of the underlying genetic mechanisms.

Ca^2+^ plays an indispensable role in the progression of cardiomyopathy. For instance, Ca^2+^/calmodulin-dependent protein kinase II and nuclear factor-κB are key players in the pathogenesis of doxorubicin-induced cardiomyopathy. Their activities are regulated by intracellular Ca^2+^, and studies have shown that blocking LTCCs alleviates doxorubicin-induced cardiomyocyte apoptosis by inhibiting intracellular Ca^2+^ overload and pathway activation. Therefore, LTCCs blockers may be potential therapeutic agents for treating doxorubicin-induced cardiomyopathy.^[44]^ Understanding the side effects of these drugs (especially those that remain unclear) and strategies for managing them could help improve both treatment efficacy and prognosis from a different perspective.

Hypertrophic cardiomyopathy (HCM) is a genetic cardiomyopathy characterized by left ventricular hypertrophy in the absence of conditions such as hypertension.^[45]^ Lehman et al^[46]^ demonstrated in a mouse model that activated calmodulin-dependent protein kinase II played a highly specific and mutation-dependent role in the progression of HCM, providing a precise therapeutic target in select cohorts. Therefore, further exploration of the relationships between different cells, enzymes, and ions could clarify the connections between mutated and normal genes, facilitating a better understanding of the mechanisms involved. This research could also enhance studies on normal physiological processes using mutations as a starting point.

In conclusion, LTCCBs exhibit significant associations with a wide range of CVDs, as evidenced by the multiple studies discussed. Detailed research findings are summarized in “Table 1,” refer to section “Table S1 Search terms, Supplemental Digital Content, https://links.lww.com/MD/P212” for “Table 1.” Future research should focus on integrating these targets through sequential or parallel approaches, which is crucial for realizing the full therapeutic potential of drugs.

**Table 1:** Confirmatory studies investigating the association between LTCCBs and cardiovascular diseases.

References	Categories	Research subject(s)	Observation indicator(s)
[19]	Hypertension	51 chronic dialysis patients who experienced an increase in systolic blood pressure (SBP) ≥ 10 mm Hg during hemodialysis without fluid overload	Changes in the increase of SBP during hemodialysis before and after the 12-week intervention
[21]	Hypertension	Wistar–Kyoto (WKY) rats and spontaneously hypertensive rats (SHR)	Investigating the effects of hydroxysafflor yellow A on blood pressure, vascular relaxation, intracellular Ca^2+^ transients, and membrane ion channels
[22]	Hypertension	SHR and WKY rats	The effect of hydrogen sulfide on enhancing the baroreceptor reflex in isolated carotid sinus
[23]	Hypertension	TGR(hAGT)L1623 cardiomyocytes and Sprague-Dawley cardiomyocytes	Intracellular activation of ICa-L mediated by the Angiotensin-(1-12)/chymase axis.
[24]	Hypertension	Normotensive rats (Wistar and WKY rats and SHR)	Evaluation of the vascular effects and mechanism of action of Hibiscus sabdariffa (HS) aqueous fraction (AF2) on isolated mesenteric arteries rings, HS AF2’s effect on ICa-L, and the extraction and enrichment of AF2 while analyzing its yield.
[25]	Hypertension	Rats	Intracellular Ca^2+^ concentration in vascular smooth muscle cells.
[30]	Arrhythmia	Isolated rabbits	Effects of isoliensinine on transmembrane ionic currents and action potentials in isolated rabbit left ventricular myocytes
[33]	Arrhythmia	Patients with symptomatic, sustained paroxysmal supraventricular tachycardia	Time to conversion to sinus rhythm during the 5-hour observation period
[34]	Arrhythmia	The Netherlands included 2503 out-of-hospital cardiac arrest (OHCA) patients and 10,543 non-OHCA controls, while Denmark included 8101 OHCA patients and 40,505 non-OHCA controls	Whether nifedipine and/or amlodipine (commonly used dihydropyridine drugs) are associated with an increased risk of OHCA, and the effects of these drugs on cardiac electrophysiology
[38]	Ischemia–reperfusion injury	SHR	Left ventricular remodeling-related indicators
[39]	Ischemia–reperfusion injury	Primary neonatal rats	The role of BAG3 in the effect of Calenduloside E on alleviating myocardial ischemia-reperfusion injury
[40]	Ischemia–reperfusion injury	Isolated rats	LTCCs, Ca^2+^ transients, and myocardial contractility
[41]	Ischemia–reperfusion injury	Rats	Cardiac-specific parameters defining heart health and early subacute myocardial infarction for all groups
[46]	Cardiomyopathy	Two different sarcomeric HCM mouse models (cardiac troponin T R92L and R92W)	Measurement of cardiac function and left ventricular remodeling, atrial remodeling monitoring, and sarcoplasmic reticulum Ca^2+^ ATPase activity

LTCCBs = L-type calcium channel blockers.

## 4. Limitations and characteristics of LTCCBs applications

As LTCCBs are a subclass of CCBs, the limitations observed in the general application of CCBs provide insight into areas where LTCCBs still require improvement. Dihydropyridine (DHP)-based CCBs are generally preferred in the long-acting formulation, as short-acting CCBs, while effective in lowering blood pressure, often accelerate heart rate and increase myocardial oxygen consumption. Common side effects include palpitations, flushing, headache, and lower limb edema, with occasional gum hyperplasia. Non-DHP CCBs, on the other hand, are typically used as first-line treatment for patients with chronic stable angina; however, due to their inhibitory effects on cardiac contractility and conduction, they are contraindicated in patients with second- or third-degree AV block and heart failure (HF). Additionally, their combined use with β-blockers can exacerbate conduction block and reduce myocardial contractility, necessitating cautious monitoring.^[47]^

### 4.1. Peripheral edema as a common adverse effect of CCBs

Peripheral edema is considered one of the common adverse effects associated with CCBs, believed to be secondary to small arterial dilation, which increases capillary pressure and leads to fluid extravasation into the interstitial space, resulting in edema. Makani et al confirmed that the incidence of peripheral edema gradually increases with the duration of CCBs treatment, up to 6 months. In the long term, more than 5% of patients discontinue CCBs use due to this adverse effect. Both non-DHP CCBs and lipophilic DHP CCBs have lower incidence rates of edema.^[48]^ Among CCBs, particularly DHP drugs such as nifedipine, a common adverse effect is vasodilatory edema. Newer CCBs, such as mibefradil, exhibit similar antihypertensive efficacy but seem to reduce the tendency to cause edema.^[49]^ To address this issue, some studies have confirmed that in hypertensive patients, combination therapy with CCBs and renin-angiotensin system blockers reduces the risk of peripheral edema associated with CCBs compared to monotherapy. Angiotensin-converting enzyme inhibitor (ACEI) appear to be more effective than Ang II receptor blocker (ARB) in reducing CCBs-related peripheral edema, though further comparative studies are required to substantiate this.^[50]^ However, attention should be given to the issue of “prescription cascade,” which refers to the phenomenon where an adverse reaction to 1 drug leads to the prescription of another drug to address those side effects, which in turn may cause new adverse effects, triggering a cycle of prescriptions. This issue is more prevalent in patients initially prescribed high-dose medications, amlodipine, or a limited range of antihypertensive drugs, particularly in male patients.^[51]^

### 4.2. Mechanisms of non-DHP CCBs and clinical medications

From a mechanistic perspective, non-DHP CCBs exhibit negative inotropic and negative chronotropic effects. These drugs can decrease myocardial contractility, improve ventricular filling, alleviate myocardial ischemia, and reduce left ventricular hypertrophy. Drugs like verapamil and diltiazem delay AV node conduction, increase the AV node refractory period, and are used in the treatment of hemodynamically stable SVT or recurrent SVT that cannot be terminated by adenosine or vagal maneuvers. However, in patients with pre-excitation syndrome and accessory pathway-mediated paroxysmal SVT, non-DHP CCBs may shorten the refractory period of the accessory pathway, resulting in an accelerated ventricular rate and potentially triggering ventricular fibrillation or hypotension, thus making them contraindicated in these cases.^[52]^ Non-DHP CCBs also have significant negative chronotropic effects. In patients with heart block or sick sinus syndrome who are hypertensive, caution is advised when using verapamil or diltiazem.^[53]^ For hypertensive patients with an elevated resting heart rate, especially those with coexisting coronary artery disease, HF, aortic dissection, or rapid atrial fibrillation (with increased ventricular rate), medications that lower blood pressure and control heart rate, such as beta-blockers, should be considered. In patients who cannot tolerate beta-blockers, non-DHP CCBs may be used (though these should be avoided in HF patients with reduced ejection fraction).^[54]^ CCBs selectively block voltage-dependent calcium channels in cell membranes, reducing myocardial contractility and causing peripheral vasodilation, which in turn lowers blood pressure. They are advantageous due to their marked antihypertensive effects, rapid onset of action, and prolonged duration. However, they are contraindicated in patients with congestive HF, as they may exacerbate HF, worsen cardiac dysfunction, and accelerate disease progression. Additionally, they can induce gastrointestinal and metabolic abnormalities, and should be avoided in patients with liver dysfunction or poor circulatory function.^[55]^ Most CCBs (except amlodipine and felodipine) have negative inotropic effects, which may lead to decompensated HF and increased mortality, and therefore, their use should be avoided.^[56]^

### 4.3. Hypertension treatment in Black and Asian populations

Helmer et al^[57]^ suggested that ACEI or ARB should not be used as first-line monotherapy for hypertension in Black patients. Instead, their use in combination with CCBs or thiazide diuretics has been found to be more effective in this population. Elevated SBP increases the risk of CVDs, mortality, and kidney function decline. In Asian populations, lifestyle factors and other factors, such as excessive salt intake and greater sensitivity to weight gain, may exacerbate the increase in SBP, leading to higher morbidity compared to Western populations. Therefore, effective SBP reduction is particularly important for Asians. Systolic hypertension in Asian populations is primarily caused by arterial stiffness. While CCBs can effectively lower peripheral blood pressure, their ability to improve arterial stiffness is limited, which may result in suboptimal control of SBP. Despite the availability of many antihypertensive drugs, blood pressure control remains inadequate in these populations.^[58]^ Tran et al^[59]^ conducted a meta-analysis and found no evidence suggesting that DHP CCBs are more effective than other antihypertensive medications in treating hypertension in Asian populations. To effectively lower blood pressure in these patients, some studies have indicated that ACEI may provide additional protection to endothelial function by inhibiting the renin-angiotensin system and reducing inflammatory markers [such as interleukin-6 and C-reactive protein]. This makes ACEI particularly suitable for treating systolic hypertension related to arterial stiffness. For instance, perindopril has shown long-lasting antihypertensive effects and reduced cardiovascular mortality risk in Asian populations.^[60]^

### 4.4. Efficacy and safety of CCBs in chronic kidney disease with hypertension

The use of CCBs, either as monotherapy or in combination with other cardiovascular drugs, demonstrates varying degrees of effectiveness in the treatment of chronic kidney disease complicated by hypertension. Among the combinations, CCBs combined with ACEI or ARB provide the best therapeutic outcomes, with the lowest incidence of adverse effects. However, the antihypertensive efficacy and adverse effect profiles of CCBs show considerable interindividual variability, influenced by genetic polymorphisms in genes such as *CACNA1C*, *CACNB2*, *CYP3A4*, *CYP3A5*, and *ABCB1*, as well as factors like gender. Additionally, the treatment effects of CCBs are also associated with the timing of administration.^[61]^ This highlights the importance of personalized medicine in clinical practice. Furthermore, the use of CCBs is associated with an increased risk of HF. As such, long-acting CCBs can be safely used for the treatment of hypertension and angina, though their protective effects on HF are less pronounced compared to other antihypertensive medications.^[1]^ Future research should continue to explore ways to mitigate the risks associated with polypharmacy in patients with multiple comorbidities, thereby enhancing patient outcomes.

## 5. Summary and outlook

This review integrates numerous research findings to summarize how LTCCBs play important roles in the diagnosis and treatment of various CVDs. Simultaneously, an in-depth examination was performed on the various scenarios of drug application, along with an identification of potential areas for enhancement, to foster the optimization of clinical drug application in future contexts. Expanding knowledge of related molecular biological pathways and clarifying unexplored pathways to improve the combined efficacy of these drugs is essential for enhancing the therapeutic effects of drug treatments (e.g., long-acting, medium-acting, and short-acting formulations). A growing body of evidence suggests that further research is needed to confirm the specific efficacy and safety of these therapies, ensuring better adherence among patients during treatment.

Moreover, the development of new LTCCBs should be accelerated to address increasingly complex disease variations. Establishing connections between natural herb-based compounds and clinically applied drugs, along with elucidating their mechanisms and targets, could expedite the development of novel drugs. Additionally, quantifying the inhibitory effects of these drugs could facilitate a new systematic classification for both existing and emerging drugs. Large-scale clinical trials on synthetic drugs of this class should be conducted, and after clarifying the molecular and genetic pathways involved, further exploration of how to optimally combine clinically applied drugs with new agents would be meaningful for enhancing therapeutic outcomes. In the future, large clinical trials could explore innovations in the pathways of normal gene functions, using point mutation genes as a starting point, which would be of even greater significance.

## Acknowledgments

We extend our thanks to all authors for their collaborative efforts.

## Author contributions

**Conceptualization:** Xiao Yang.

**Methodology:** Xiao Yang.

**Resources:** Xiao Yang.

**Supervision:** Xinghui Li.

**Writing – review & editing:** Xiao Yang.

## Supplementary Material


