# Supplementary material for: Advances in research on the correlation between LTCCBs and cardiovascular diseases: A review
Source: Medicine (Baltimore). 2025 Jun 20;104(25):e42799. doi: 10.1097/MD.0000000000042799 (PMC12187283; doi:10.1097/MD.0000000000042799)
Supplement: Supplementary file 1 [file medi-104-e42799-s001.docx]

SHR = spontaneously hypertensive rats, HS = hibiscus sabdariffa, AF2 = aqueous fraction, OHCA = out-of-hospital cardiac arrest.
